# Metagenome-assembled genomes from microbial communities in lab-scale anaerobic bioreactors treating simulated dairy wastewater

**DOI:** 10.1128/mra.00487-25

**Published:** 2025-11-14

**Authors:** Simon Mills, Gavin Collins, Umer Zeeshan Ijaz, Piet N. L. Lens

**Affiliations:** 1 School of Biological and Chemical Sciences, and The Ryan Institute, University of Galway 8799, Galway, Ireland; 2 Water & Environment Research Group, University of Glasgow, Mazumdar-Shaw Advanced Research Centre 3526, Glasgow, United Kingdom; 3 Department of Molecular and Clinical Cancer Medicine, University of Liverpool 105727, Liverpool, United Kingdom; Montana State University, Bozeman, Montana, USA

**Keywords:** anaerobic digestion, metagenome-assembled genomes, methanogens

## Abstract

This dataset describes metagenome-assembled genomes from three lab-scale (4L) expanded granular sludge bed bioreactors treating synthetic dairy wastewater. The resulting MAGs encompass 60 phyla with average genome completeness of 78.68% and average contamination of 2.29%. These genomes represent a valuable resource for studying anaerobic bioreactors used in wastewater treatment.

## ANNOUNCEMENT

During anaerobic digestion, organic matter is degraded by microbial communities in the absence of oxygen, leading to the formation of biogas. These communities are susceptible to environmental disturbances that can impair syntrophic interactions and lead to process failure (1, 2).

Three lab-scale (4 L) expanded granular sludge bed (EGSB) anaerobic bioreactors were inoculated with granular sludge from a mesophilic (37°C) internal circulation bioreactor (650  m³) treating dairy and ethanol-production wastewater in Ballineen, Ireland (OLR: 9.5 kg COD/m³/d; HRT: 21  h). The EGSBs were fed skimmed milk powder with minerals and trace metals and operated at HRT 18  h, OLR 4.6 g COD/L/day, each under a different disturbance regime followed by an organic shock. Triplicate granular sludge samples were collected (Lat: 53.2793, Long: −9.0586), aliquoted into 1.5  mL tubes, snap frozen in liquid nitrogen, and stored at –80°C. Full experimental details are published elsewhere (3, 4).

DNA was extracted using a phenol-chloroform method (5). Stored samples were crushed in microcentrifuge tubes for a homogeneous mix; 0.2 g was added to bead tubes (Macherey-Nagel, Type A, Fisher Scientific, Dublin, Ireland). Cells were lysed by bead beating in 1% CTAB lysis buffer followed by phenol-chloroform extraction. DNA concentration was quantified using a Qubit High-Sensitivity kit (Invitrogen, Carlsbad, CA, USA) and quality checked via agarose gel electrophoresis and NanoDrop. Library preparation and sequencing were performed by Novogene (Cambridge, UK), including fragmentation, end-repair, phosphorylation, A-tailing, and adaptor ligation. Paired-end 150 bp sequencing was done on an Illumina NovaSeq 6000 with SP Reagent Kit v1.5.

Quality trimming was done using Sickle v1.200 (6) resulting in average Phred scores of <20. Any reads <50 bp were discarded, resulting in 641,256,041 reads. Co-assembly was performed using Megahit v1.1.3 (7) with the parameters --k-list 27,47,67,87 --kmin-1pass -m 0.95 --min-contig-len 1000 28, resulting in 653,274 contigs. Contigs were binned with MetaWrap v1.3.2 (8), using three different binning algorithms: metabat2 v2.12.1 (610 bins), maxbin2 v2.2.4 (520 bins), and CONCOCT v1.1.0 (381 bins). Default parameters were used, except where otherwise noted.

**Fig 1:**
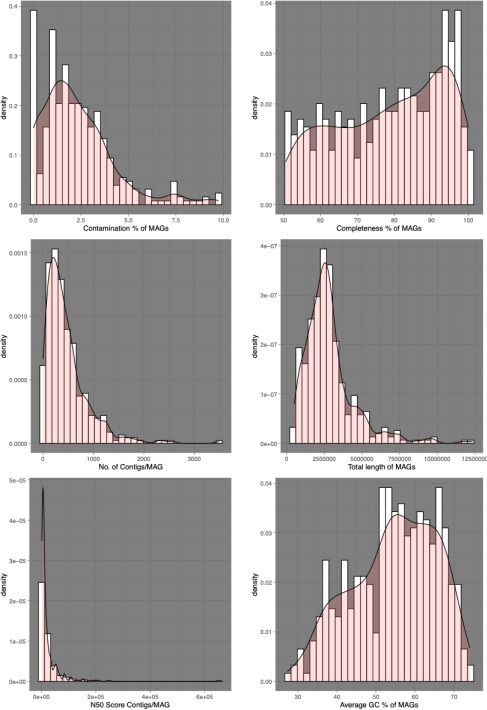
Density plots showing distributions of MAG completeness, contamination, N50, and GC content across 380 bins.

CheckM v1.0.12 (9) was used to determine the completion and contamination in each bin using default parameters. All bins with ≥50% completion and ≤10% contamination were consolidated, resulting in 380 metagenomic assembled genomes (MAGs). Recovered MAGs had a mean genome completion of 78.68%, mean contamination of 2.29%, mean length of 2,856,760 bps, mean number of contigs of 470, mean N50 score of 23,610, and mean GC content of 55% (Fig. 1). MAGs were taxonomically classified by GTDB-Tk release 95 (10), resulting in genomes from 60 phyla. A total of 356 MAGs were classified as bacteria, and 24 MAGs were classified as archaea. The METABOLIC v4.0 (11) pipeline was used to assign functions to each bin (12).

These MAGs are a numerous and diverse source of microorganisms when compared with other resource announcements from similar environments (13, 14). The most abundant bacterial phyla included *Chloroflexota* (10%), *Bacteroidota* (10%), and *Patescibacteria* (9.7%). The most abundant archaeal phylum was Halobacteriota (3.68%).

## Data Availability

Raw sequencing data can be found on the NCBIs Sequence Read Archive (SRA) under the accession number PRJEB89300 with the BioSample numbers SAMEA118255448 - SAMEA118255465. Assembled MAGs are deposited on Figshare (https://doi.org/10.6084/m9.figshare.25795075.v1).

## References

[1] Amha YM, Anwar MZ, Brower A, Jacobsen CS, Stadler LB, Webster TM, Smith AL. 2018. Inhibition of anaerobic digestion processes: applications of molecular tools. Bioresour Technol 247:999–1014. doi:10.1016/j.biortech.2017.08.210 28918349

[2] Saha S, Basak B, Hwang J-H, Salama E-S, Chatterjee PK, Jeon B-H. 2020. Microbial symbiosis: a network towards biomethanation. Trends Microbiol 28:968–984. doi:10.1016/j.tim.2020.03.012 33171105

[3] Mills S, Ijaz UZ, Lens PNL. 2025. Environmental instability reduces shock resistance by enriching specialist taxa with distinct two component regulatory systems. NPJ Biofilms Microbiomes 11:1–15. doi:10.1038/s41522-025-00679-w 40164638 PMC11958701

[4] Mills S, Yen Nguyen TP, Ijaz UZ, Lens PNL. 2023. Process stability in expanded granular sludge bed bioreactors enhances resistance to organic load shocks. J Environ Manage 342:118271. doi:10.1016/j.jenvman.2023.118271 37269726

[5] Griffiths RI, Whiteley AS, O’Donnell AG, Bailey MJ. 2000. Rapid method for coextraction of DNA and RNA from natural environments for analysis of ribosomal DNA- and rRNA-based microbial community composition. Appl Environ Microbiol 66:5488–5491. doi:10.1128/AEM.66.12.5488-5491.2000 11097934 PMC92488

[6] Joshi N, Fass J. 2011. Sickle: a sliding-window, adaptive, quality-based trimming tool for FastQ files (Version 1.33)

[7] Li D, Liu C-M, Luo R, Sadakane K, Lam T-W. 2015. MEGAHIT: an ultra-fast single-node solution for large and complex metagenomics assembly via succinct de Bruijn graph. Bioinformatics 31:1674–1676. doi:10.1093/bioinformatics/btv033 25609793

[8] Uritskiy GV, DiRuggiero J, Taylor J. 2018. MetaWRAP—a flexible pipeline for genome-resolved metagenomic data analysis. Microbiome 6:158. doi:10.1186/s40168-018-0541-1 30219103 PMC6138922

[9] Parks DH, Imelfort M, Skennerton CT, Hugenholtz P, Tyson GW. 2015. CheckM: assessing the quality of microbial genomes recovered from isolates, single cells, and metagenomes. Genome Res 25:1043–1055. doi:10.1101/gr.186072.114 25977477 PMC4484387

[10] Chaumeil P-A, Mussig AJ, Hugenholtz P, Parks DH. 2020. GTDB-Tk: a toolkit to classify genomes with the Genome Taxonomy Database. Bioinformatics 36:1925–1927. doi:10.1093/bioinformatics/btz848 PMC7703759 31730192

[11] Zhou Z, Tran PQ, Breister AM, Liu Y, Kieft K, Cowley ES, Karaoz U, Anantharaman K. 2022. METABOLIC: high-throughput profiling of microbial genomes for functional traits, metabolism, biogeochemistry, and community-scale functional networks. Microbiome 10:33. doi:10.1186/s40168-021-01213-8 35172890 PMC8851854

[12] Kanehisa M, Goto S. 2000. KEGG: kyoto encyclopedia of genes and genomes. Nucleic Acids Res 28:27–30. doi:10.1093/nar/28.1.27 10592173 PMC102409

[13] Nesbø CL, Fitamo TM, Lee H, Edwards EA. 2024. Metagenomes and metagenome-assembled genomes from a sequentially fed anaerobic digester treating solid organic municipal waste. Microbiol Resour Announc 13:e0091923. doi:10.1128/mra.00919-23 38126755 PMC10793357

[14] Walters KA, Myers KS, Wang H, Fortney NW, Ingle AT, Scarborough MJ, Donohue TJ, Noguera DR. 2022. Metagenomes and metagenome-assembled genomes from microbial communities fermenting ultrafiltered milk permeate. Microbiol Resour Announc 11:e0029322. doi:10.1128/mra.00293-22 35770995 PMC9302107

